# Functional Bias and Spatial Organization of Genes in Mutational Hot and Cold Regions in the Human Genome

**DOI:** 10.1371/journal.pbio.0020029

**Published:** 2004-02-17

**Authors:** Jeffrey H Chuang, Hao Li

**Affiliations:** **1**Department of Biochemistry and Biophysics, University of CaliforniaSan Francisco, CaliforniaUnited States of America

## Abstract

The neutral mutation rate is known to vary widely along human chromosomes, leading to mutational hot and cold regions. We provide evidence that categories of functionally related genes reside preferentially in mutationally hot or cold regions, the size of which we have measured. Genes in hot regions are biased toward extracellular communication (surface receptors, cell adhesion, immune response, etc.), while those in cold regions are biased toward essential cellular processes (gene regulation, RNA processing, protein modification, etc.). From a selective perspective, this organization of genes could minimize the mutational load on genes that need to be conserved and allow fast evolution for genes that must frequently adapt. We also analyze the effect of gene duplication and chromosomal recombination, which contribute significantly to these biases for certain categories of hot genes. Overall, our results show that genes are located nonrandomly with respect to hot and cold regions, offering the possibility that selection acts at the level of gene location in the human genome.

## Abstract 2

Functionally related genes tend to be found in regions of the genome with high or low mutation rates, which implies that natural selection can also operate at the level of gene location

## Introduction

Because of the abundant availability of mouse and human genome data (International Human Genome Sequencing Consortium 2001; Mouse Genome Sequencing Consortium 2002), it has come to light that mutation rates vary widely across different regions of the human genome (Matassi et al. 1999; Mouse Genome Sequencing Consortium 2002; Hardison et al. 2003), in agreement with a number of smaller-scale studies (Wolfe et al. 1989; Casane et al. 1997; Perry and Ashworth 1999). Regions of unusually high or low substitution rates have been observed from 4-fold sites and ancestral repeat sequences, two of the best candidates for measuring neutral rates of mutation in mammals (Sharp et al. 1995; Mouse Genome Sequencing Consortium 2002; Hardison et al. 2003). The reasons for such regional variability are unclear, since structural characterizations of the mutation rate are nascent. Whatever the reason for these hot and cold regions, their existence suggests a question that has intriguing consequences for molecular evolution: does the organism take advantage of these hot and cold spots?

One way to take advantage of a hot region would be to place genes there for which the hotness is useful—an intuitive example would be receptor proteins, which must respond to a constantly changing ligand set. Similarly, it could be beneficial to place delicate genes in a cold region, to reduce the possibility of deleterious mutations. These potential advantages offer the possibility that regional mutation rates affect the spatial organization of genes. The idea of such organization in mouse and human is bolstered by recent findings of gene organization in yeast. For example, Pal and Hurst (2003) showed that yeast genes are organized to take advantage of local recombination rates, which is particularly relevant since mutation rate and recombination rate are known to be correlated (Lercher and Hurst 2002). If the local mutation rate—equivalent to the synonymous (amino acid preserving) substitution rate *K_S_* if synonymous substitutions are neutral—affects gene organization, this would constitute a type of selection complementary to traditional selection on point mutations (Graur and Li 2000).

We studied whether local mutation rates affect gene locations by measuring the mutation rates of genes and their organization in the human genome. First, we analyzed the substitution rates of the genes in each of the families defined by the Gene Ontology (GO) Consortium (Ashburner et al. 2000). If the organism is taking advantage of varying *K_S_*, gene families should be biased toward regions of appropriate rate. In fact, we observe that several functional classes of genes preferentially occur in hot or cold regions. Some of the notable hot categories we observe are olfactory genes, cell adhesion genes, and immune response genes, while the cold categories are biased toward regulatory proteins such as those involved in transcription regulation, DNA/RNA binding, and protein modification. Also, to better characterize the hot and cold regions, we measured the length scale over which substitution rates vary. While rough limits on the size of hot and cold regions are known (Matassi et al. 1999; Hardison et al. 2003), this paper presents the first known quantitative calculation of their length scale.

Because mutation rates are regional, mutation rates in genes categories could be influenced by events altering the organization of genes in the genome, such as gene relocation or gene duplication. We therefore analyzed mechanisms by which functional categories of genes may have become concentrated in hot or cold regions. A clustering analysis reveals that the hotness of some categories is enhanced by local gene duplications in hot regions. However, there are strong functional similarities among the hot categories—both clustered and unclustered—as well as among the cold categories. These functional similarities imply that the instances of duplicated categories are not random; i.e., selection may have affected which genes have duplicated and persisted.

## Results

### Mutation Rates Have Regional Biases

Recently, substitution rates between Mus musculus and Homo sapiens have been measured by several groups on a genome-wide scale (Kumar and Subramanian 2002; Mouse Genome Sequencing Consortium 2002; Hardison et al. 2003). These substitution rates vary significantly across the genome (Mouse Genome Sequencing Consortium 2002; Hardison et al. 2003), suggesting that neutral mutation rates may have regional biases as well. A popular proxy for neutral mutation rates is the substitution rate at 4-fold sites (a recent example is found in Kumar and Subramanian [2002]), base positions in coding DNA that do not affect protein sequence and that should hence be under less selective pressure than other sites. The 4-fold sites also offer the advantage of being easily alignable.

For these reasons, we estimated the neutral mutation rate from substitution rates at 4-fold sites (which we use interchangeably with the term *K_S_* in this paper). This identification is not without complexities, however, since there are processes that can in principle selectively affect the 4-fold sites. For example, some have argued that exogenous factors such as isochore structure influence the silent sites (Bernardi 2000), and codon usage adaptation has been shown to affect silent sites in bacteria and yeast (Sharp and Li 1987; Percudani and Ottonello 1999). So far, such selective effects have been difficult to detect in mammals (Smith and Hurst 1999a; Duret and Mouchiroud 2000; Iida and Akashi 2000; Kanaya et al. 2001). Recently, Hardison et al. (2003) showed that several functionally unrelated measures of mutation rate, including SNP density, substitutions in ancestral repeats, and substitutions in 4-fold sites, are correlated in genome-wide mouse–human comparisons—suggesting that these measures have common neutral aspects.

We constructed our own dataset of the 4-fold substitution rates for 14,790 mouse/human orthologous genes, using data from the ENSEMBL consortium. In order to properly account for stochastic finite-size effects, we mapped the observed substitution rates to a normalized value, based on the assumption that all 4-fold sites mutate at the same rate (see Materials and Methods). Under this assumption, it was expected that the normalized substitution rates would follow the normal distribution (a Gaussian distribution with σ = 1).

**Figure 1:**
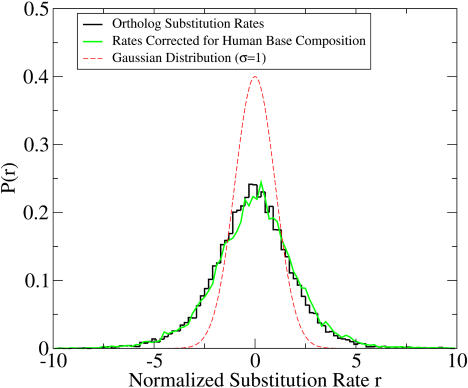
Distribution of Normalized Substitution Rates Histogram of substitution rates based on 14,790 orthologous mouse and human genes (black curve). The rate distribution has significantly more genes at high and low rates than the expected Normal distribution (red curve). This bias toward high and low rates remains even when rates are corrected for human base composition (green curve).

Contrary to these expectations, the distribution of ortholog substitution rates was found to be highly biased toward high or low rates, indicating that 4-fold mutation rates vary substantially by location and on a scale larger than the typical size of a gene. Figure 1 shows the distribution of substitution rates for all mouse/human orthologs. The observed distribution has excesses of genes at both high and low substitution rates. These results are in agreement with the findings of Matassi et al. (1999), who reported significant mutation rate correlations between neighboring genes. This is not a compositional effect—the distribution remained the same even when corrections for the gene's human base composition were made (see Materials and Methods). We further verified that substitution rates of neighboring genes were correlated using an analysis qualitatively similar to Matassi et al. (1999)—though with approximately 20 times more orthologs—finding that gene substitution rates are correlated with their neighbors with a *p*-value of 10^−189^ (see Materials and Methods). These results imply that substitution rates have regional biases, acting both within a gene and over longer length scales.

### Some Gene Categories Are Biased toward Hot or Cold Regions

We next considered whether there is a relationship between gene locations and their functions, i.e., whether functional categories of genes have biases for being in regions of particular mutation rate. To test whether such biases exist, we performed an analysis of the GO assignments for each ortholog pair (Ashburner et al. 2000), using data from the ENSEMBL human ENSMART database to assign genes to GO categories. For each GO category, we calculated a *z*-score to measure the overall substitution rate, based on the substitution rates of the genes in the category (see Materials and Methods). The 21 GO categories having statistically significant positive values of *z* are shown in Table 1. In terms of 4-fold substitution rates, the hot category rate averages were found to range from 0.346 (integral to membrane) to 0.468 (internalization receptor activity), while the genome-wide average was 0.337 (with a genewise standard deviation of 0.08). For a category with several genes, the effective standard deviation is much smaller, equal to 0.08/√*N_GO_* , where *N_GO_* where *N_GO_* is the number of genes in the category, so these rate biases are extremely significant. Hot gene categories were focused mainly in receptor-type functions, along with a few other categories such as “proteolysis” and “microtubule motor activity.” Some preferences were partially because categories have genes in common; e.g., eight genes are shared among the categories “dynein ATPase activity,” “dynein complex,” and “microtubule-based movement.” However, there were several categories of similar function that were independent; e.g., “membrane” and “olfactory receptor activity” shared no genes, and “cell adhesion” and “immune response” shared only 5% of their genes. Overall, there was a clear bias for the larger hot categories to contain receptor-type proteins: e.g., “receptor activity,” “olfactory receptor activity,” “G-protein coupled receptor protein signaling pathway,” “membrane,” and “immune response.” For the set of all 1,488 genes where the string “receptor” is part of the GO description, the average 4-fold substitution rate was found to be 0.347. The probability that a random set of 1,488 genes would have an average rate this high is 10^−6^.

**Table 1:**
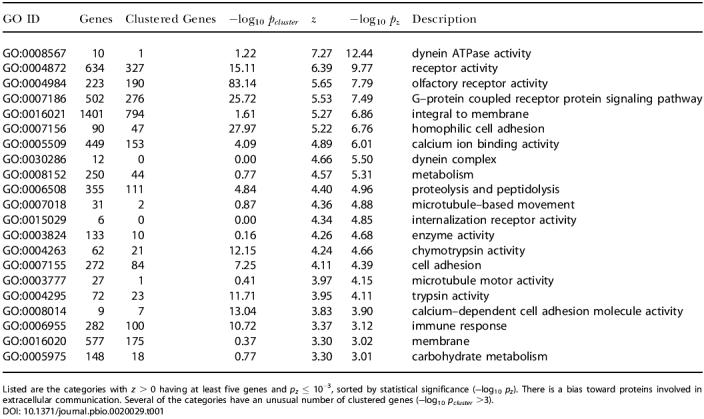
Statistically Significant Hot GO Categories

Listed are the categories with *z* > 0 having at least five genes and *p_z_* ≤ 10^−3^, sorted by statistical significance (−log_10_ *p_z_*). There is a bias toward proteins involved in extracellular communication. Several of the categories have an unusual number of clustered genes (−log_10_ *p_cluster_* >3)

The 36 statistically significant GO categories with negative *z* scores, are shown in Table 2. The 4-fold rate averages for the cold categories ranged from 0.220 (“mRNA binding activity”) to 0.326 (“protein serine/threonine kinase activity”). The coldest gene categories included “nuclear proteins,” “transcription regulation,” “DNA and RNA binding,” “oncogenesis,” “phosphatases,” and “kinases,” all of which are important to regulatory processes. Many of these genes are also housekeeping genes (Hsiao et al. 2001). For the set of all 1,704 genes where the string “regulat” is part of the GO description, the average 4-fold substitution rate was found to be 0.325. The probability that a random set of 1,704 genes would have an average rate this low is 10^−9^.

**Table 2:**
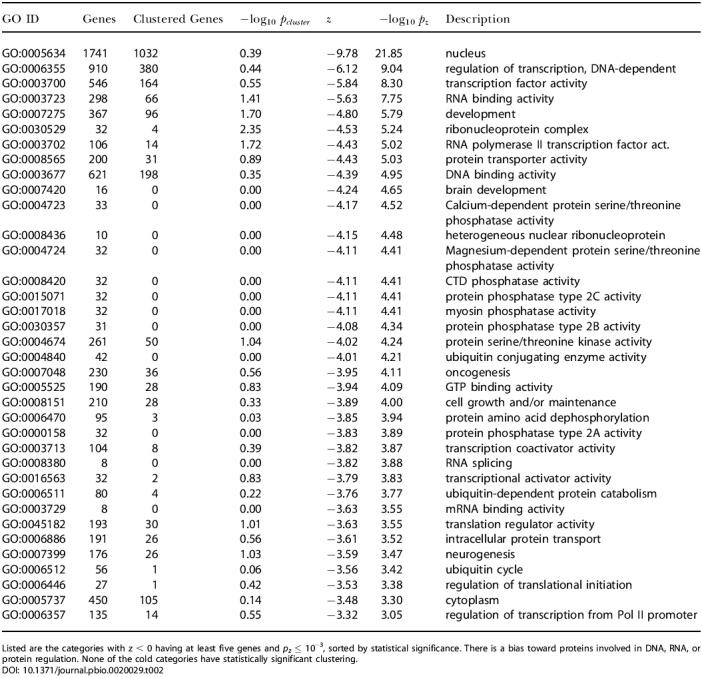
Statistically Significant Cold GO Categories

Listed are the categories with z < 0 having at least five genes and *p_z_* ≤ 10^−3^, sorted by statistical significance. There is a bias toward proteins involved in DNA, RNA, or protein regulation. None of the cold categories have statistically significant clustering

We repeated our *z*-score classifications using several other measures of mutation rate and in each case inferred similar hot and cold categories. For example, under the normalized rate model that accounts for human base composition, the same set of 23 hot categories were found. Of the 37 cold categories, 33 remained classified as cold. The four lost were “regulation of transcription from Pol II promoter,” “development,” “neurogenesis,” and “translation regulator activity.” There were six new categories, and these were also largely regulatory: “nucleic acid binding activity,” “translation initiation factor activity,” “ubiquitin C-terminal hydrolase activity,” “collagen,” “RNA processing,” and “negative regulation of transcription.” We also calculated several maximum likelihood (ML) measures of *K_S_* using mutation models in the Phylogenetic Analysis by Maximum Likelihood (PAML) package (Yang 1997), including the Nei and Gojobori (1986) codon-based measure and the TN93 (Tamura and Nei 1993) and REV (Tavere 1986) models. We again found qualitatively similar sets of hot and cold categories—receptor genes at high substitution rates and regulatory genes at low substitution rates—though there were changes in the numbers of significant categories. For example, for the TN93 model, we observed ten hot categories—“induction of apoptosis by extracellular signals,” “G-protein coupled receptor protein signaling pathway,” “olfactory receptor activity,” “receptor activity,” “apoptosis,” “enzyme activity,” “chymotrypsin activity,” “trypsin activity,” “integral to membrane,” and “dynein ATPase activity”—and eight cold categories: “calcium-dependent protein serine/threonine phosphatase activity,” “ribonucleoprotein complex,” “protein serine/threonine kinase activity,” “RNA binding activity,” “protein amino acid dephosphorylation,” “intracellular protein transport,” “protein transporter activity,” and “nucleus.” The categories inferred from our original *z*-score analysis are probably more accurate than those from ML methods, because ML methods tend to produce strong outliers at high substitution rate, skewing calculations of the variance in the *z*-score analysis.

### Can Gene Duplications Explain the Hot and Cold Categories?

Given the existence of hot and cold gene categories, the question then becomes: why do these biases exist? One potentially nonselective factor that could affect category rate biases is local gene duplications. New genes generally arise by duplication, in which a new copy of a gene is generated nearby to the preexisting gene by a recombinatorial event such as unequal crossing-over, followed by evolution to a novel, but often related function (Graur and Li 2000). Such local duplications can cause many genes with similar function to be clustered together. Because there are regional biases in mutation rate (discussed in the section on Block Structure of the Substitution Rate), these functionally related genes will tend to have similar mutation rates. GO categories containing these genes will then be biased toward the mutation rate of the region surrounding the genes.

We tested the effect of gene duplications on category rates through a clustering analysis (see Materials and Methods). If gene duplications are not important to category rates, genes in a hot (cold) gene category would be expected to be distributed randomly throughout the many hot (cold) regions around the genome; i.e., clustering of genes would be weak. However, if gene duplications are relevant, we would expect hot (cold) genes of the same category to be tightly clustered since many of these genes would have arisen by local duplications. We therefore studied the location distribution of each of the gene categories and analyzed the significance of its clustering, using the short-range correlation length τ ∼ 10^6^ basepairs (see the section on Block Structure of the Substitution Rate) as a defining length scale. This analysis was similar to that of Williams and Hurst (2002), who studied clustering of tissue-specific genes, though we analyzed a larger number of more narrowly defined gene families.

**Figure 2:**
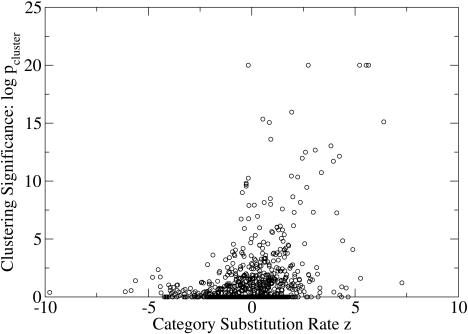
Clustering versus Substitution Rate for GO Categories Containing at Least Five Members Virtually all clustered gene categories have higher than average substitution rates (*z* > 0).

We found that some of the hot gene categories were indeed clustered, but that none of the cold gene categories were. The results of the clustering for the hot and cold categories are displayed in Tables 1 and 2, with the clustering p-values shown via their −log_10_ values. Of the 21 statistically significant hot categories, ten categories had statistically significant clustering (−log_10_ *p_cluster_* > 3). For example, the “olfactory receptor activity” category has 223 genes, with a randomly expected number of clustered genes equal to 30.6. The actual number of clustered genes was found to be 190, which has a *p*-value of less than 10^−16^. In the set of 37 cold gene GO categories, none had statistically significant clustering. The clustering significance is plotted versus the substitution score *z* for all the GO categories with at least five members in Figure 2. There were many categories of hot genes with significant clustering (−log_10_ *p_cluster_* > 3), but virtually no cold ones.

**Figure 3:**
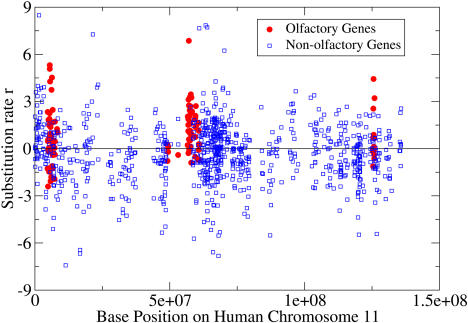
Clustering of Olfactory Genes on Human Chromosome 11 The olfactory genes are clustered into three regions along the chromosome. The substitution rates of the olfactory genes are almost all hot, while the nonolfactory genes are distributed around *r* = 0.

**Figure 4:**
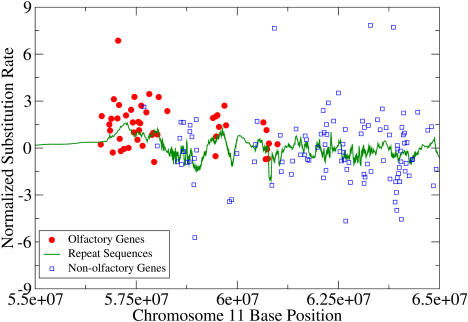
Olfactory Genes Lie in a Mutational Hot Spot Substitution rates of the olfactory genes in the central region of human Chromosome 11. The substitution rate of ancestral repeat sequences is higher in the region where the olfactory genes lie.

As an example of clustering in the hot gene categories, we considered the olfactory receptors. It is well-established that olfactory receptors occur in clusters throughout the human genome (Rouquier et al. 1998), and we likewise observed the olfactory receptors to be highly clustered in three regions near the head, middle, and tail of Chromosome 11 (Figure 3). The central cluster is displayed in Figure 4. This clustering provided evidence that local gene duplications have influenced the high category rate of the olfactory genes.

We next attempted to determine whether the high olfactory rates are due to a regional bias. The substitution rates of all genes are plotted in Figure 4, with the olfactory genes in red. As expected, the olfactory genes exhibited an obvious bias for higher substitution rates than other genes. We next calculated the mutation rate of the region as determined from an independent measure, the substitution rates between ancestral repeat sequences (green curve in Figure 4), using data published by Hardison et al. (2003) (see Materials and Methods). The repeat sequence mutation rate was notably higher in the regions where the olfactory genes occur, showing that the hotness of the olfactory genes is a regional property and not specific to the genes.

**Figure 5:**
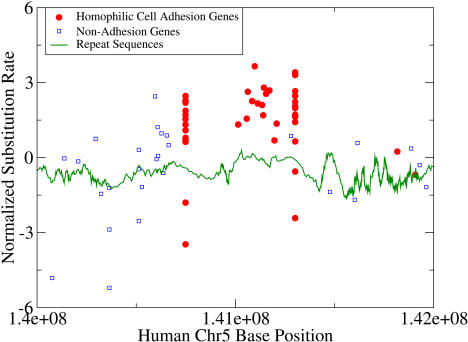
Homophilic Cell Adhesion Genes Also Lie in a Hot Spot Substitution rates of a cluster of homophilic cell adhesion genes on human Chromosome 5, along with substitution rates of other genes and ancestral repeat sequences. The repeat sequence substitution rate plateaus at a higher level in this region.

Similar clustering and regional hotness were observed for other hot gene categories. We plot the substitution rates of a cluster of homophilic cell adhesion genes on Chromosome 5 in Figure 5, along with the rates of nearby genes and the ancestral repeat sequence substitution rates. The same features observed for the olfactory genes were also present for the cell adhesion genes: clustering, high substitution rates, and an elevated ancestral repeat substitution rate. The repeat substitution rate exhibited a plateau-like behavior over the region defined by the homophilic cell adhesion genes. These factors support the interpretation that significant numbers of hot genes have arisen by duplications in inherently hot regions of the genome.

### Block Structure of the Substitution Rate

Several explanations have been proposed that could account for the regional biases in mutation rate (Mouse Genome Sequencing Consortium 2002), including recombination-associated mutagenesis (Perry and Ashworth 1999; Lercher and Hurst 2002), strand asymmetry in mutation rates (Francino and Ochman 1997), and inhomogeneous timing of DNA replication (Wolfe et al. 1989; Gu and Li 1994). The structure of regional biases could be considered from the perspective of amino acid changing substitutions as well, since linked proteins have been known to have similar substitution rates (Williams and Hurst 2002). However, the silent sites may be easier to comprehend, since protein sequences are more likely to be complicated by nonneutral pressures.

**Figure 6:**
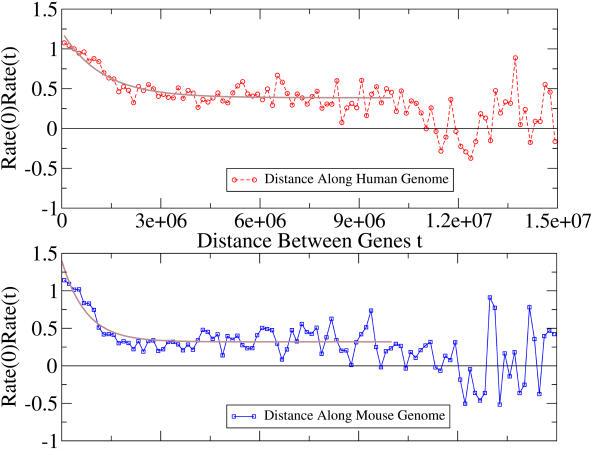
Correlation Length Analysis of Substitution Rates Correlation of substitution rates in syntenous blocks as a function of distance between genes measured along the human chromosome (top) and measured along the mouse chromosome (bottom). There are two length scales of correlation decay: a short one of 1 Mb and a long one of 10 Mb. The curve fits are for <*r*(0)*r*(*t*)> = *A*_0_ exp (−*t/τ*) + *A*_∞_ for the region *t* ∈ [0, 10000000] .

To shed light on the structural properties of the hot and cold mutational regions, we measured the length scale over which substitution rates are correlated. Previously, correlations have been observed in blocks of particular physical (5 Mb) (Hardison et al. 2003) or genetic (1, 2, 5, and 200 cM) (Matassi et al. 1999; Lercher et al. 2001) size. While these studies have focused on whether correlations exist at certain length scales, it is informative to measure the decay of correlations with distance. We therefore measured the length scale of substitution rate correlation, using an analysis of the correlation function (Huang 1987)



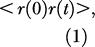
where *r*(*t*) is the substitution rate of a gene *t* basepairs downstream of a gene with substitution rate *r*(0), and <…> indicates an average over the available data (see Materials and Methods). We expect that at small *t*, the correlation function will be positive and then decrease with *t* as rates become decoupled. The length scale over which this decay occurs serves as a measure of the typical size of hot or cold regions. The rate correlation function is plotted in Figure 6 versus both the human and mouse values for *t*.

We observed two notable behaviors: (1) a strong correlation that decays over a region of approximately 1 Mb, and (2) a longer range correlation which plateaus over a region of approximately 10 Mb. At larger distances, correlations are weaker. For example, the human curve first dips below the <*r*(0)*r*(*t*)> = 0 threshold at approximately 11 Mb, and the mouse curve first crosses it at approximately 9 Mb. This suggests that there are multiple phenomena that control the mutation rate of regions, both long (10 Mb) and short (1 Mb) length scale.

We also measured the characteristic short-range correlation length using an exponential fit. The correlation length τ was determined by fitting the data to the functional form



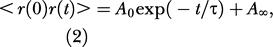
where *A*_∞_ is the correlation at long distances and (*A*_0 _+ *A*_∞_) is the correlation at zero distance. Because of the observed plateauing behavior of the data, we performed our curve fit over the region *t* ∈ [0, 10000000] . For the human data, we obtained *A*_0_ = 0.83, τ = 1.21 × 10^6^, *A*_∞_ = 0.39. For the mouse data, we found values of a similar magnitude (*A*_0_ = 1.08, τ = 0.73 × 10^6^, *A*_∞_ = 0.32), suggesting that short-range mutational processes may be alike in mouse and human. The long-range correlation *A*_∞_ was at least an order of magnitude larger than would be expected by chance at all distances up to 10 Mb (see Materials and Methods).

It is unclear what factors are responsible for these two length scales of rate correlation, though some guesses are possible. For the short-range effect, one process that occurs on the appropriate length scale is DNA replication (Alberts et al. 1994). Replication origins in a concerted unit activate under similar timing and similar cell conditions and could have a common regulatory mechanism, making it a reasonable to expect the DNA in such a unit to have similar mutation rates.

Long-range correlations have previously been observed at chromosomal-size distances in particular regions of the genome; e.g., it is known that Chromosome 19 is generally hotter than other chromosomes (Lercher et al. 2001; Castresana 2002). However, the 10 Mb correlation was not simply due to selection on chromosomes. We removed the respective chromosomal average from each substitution rate and repeated the correlation analysis, finding that *A*_∞_ retained a significant value of approximately 0.2. One possible mechanistic explanation for the long-range correlation is suggested by the finding of Lercher and Hurst (2002) that recombination rate and substitution rate are correlated even in blocks extending to 30 Mb. Therefore, if large regions of similar recombination rate exist, they could be related to the long-range 4-fold correlation effects we observed.

## Discussion

### Evidence for Selection

Recently, there has been evidence for selective factors influencing gene location in yeast (Pal and Hurst 2003). This suggests the possibility that similar phenomena affect gene locations in mouse/human as well. We therefore considered whether regional mutation rates could have selectively influenced the types of genes occurring in different loci in the genome. Selection due to the local mutation rate would require different mechanisms than that observable through the traditional measure *K_A_/K_S_*, which quantifies selection on point mutations. For example, regional mutation rates could have influenced the fitness of the genome after events that cause gene relocation, such as gene transposition or chromosomal recombination. Or perhaps the duplication of certain genes provided a fitness benefit (a mechanism possibly relevant for the hot clustered categories). Differential duplication rates could force a category to have a mutation rate bias, due to the block structure of the mutation rate and the fact that duplications occur locally.

The observed categories of hot and cold genes suggest gene locations have been selectively influenced by regional mutation rates. This is because if mutation rates were unrelated to gene function, then the lists of hot and cold categories would be expected to be random; i.e., the lists shown in Tables 1 and 2 would have been evenly sampled from all possible GO categories. However, this was not the case, as the hot and cold categories each had strong internal commonalities.

The hot categories were found to be biased toward receptor activities or roles in extracellular communication. Intriguingly, arguments based on protein-level effects appear applicable to the silent-site hotness of these categories. Cellular receptors and those involved in extracellular communication are the proteins that most directly interact with the environment and are therefore the most likely to have experienced a dynamically changing set of selection pressures. This variability of selection pressures would have made it favorable for them to be in hot regions, in order that new mutations be possible to deal with new stimuli. Examples of hot categories with known protein-level diversification pressures include the olfactory receptors (Lane et al. 2001), immune genes (Papavasiliou and Schatz 2002), and cell adhesion genes (Uemura 1998; Tasic et al. 2002).

Arguments normally applied to protein-level selection were found to be appropriate for cold mutation rate categories as well. Cold categories were often related to transcription or other regulatory processes. Regulatory proteins should be tuned to interact with many different nucleic acid or protein targets, in contrast with receptor proteins, which typically interact with only a particular ligand. Mutations to regulatory proteins would therefore be expected to be more deleterious, and hence it would be beneficial for them to have low mutation rates. Strong conservation pressures in the cold categories could also be related to their roles as housekeeping genes (Zhang and Li 2003) or as essential genes. For example, in the dataset of Winzeler et al. (1999), 81 out of 356 essential yeast genes were involved in transcription, whereas only four were involved in signal transduction, the function most similar to extracellular communication for which data were available.

The applicability of protein-level arguments to synonymous mutation rates suggests that *K_S_* and *K_A_* are under similar pressures. This is consistent with what would be expected if gene locations have evolved to make use of the block structure of the mutation rate, since relocation to a hot (cold) spot would increase propensities for both high (low) *K_A_* and *K_S_*. More quantitatively, we observed that *K_S_* category biases were similar to category biases caused by selection on amino acid changing point substitutions—i.e., selection observable through the ratio *K_A_/K_S_*. We performed a GO *z*-score analysis on *K_A_/K_S_* (for consistency, the CODEML method in PAML was used to calculate both *K_A_* and *K_S_*). There were eight hot categories common to both the 4-fold and *K_A_/K_S_* classifications (“immune response,” “proteolysis receptor activity,” “peptidolysis receptor activity,” “integral to membrane,” “chymotrypsin activity,” “cell adhesion,” “trypsin activity,” “olfactory receptor activity”) and 17 common cold categories (“nucleus,” “regulation of transcription,” “transcription factor activity,” “RNA binding activity,” “development,” “ribonucleoprotein complex,” “protein transporter activity,” “protein serine/threonine kinase activity,” “ubiquitin conjugating enzyme activity,” “GTP binding activity,” “ubiquitin-dependent protein catabolism,” “translation regulator activity,” “intracellular protein transport,” “neurogenesis,” “ubiquitin cycle,” “cytoplasm,” “regulation of transcription from Pol II promoter”). The strong commonalities between the two types of classification suggest that the selective forces that influenced amino acid changing point mutations also influenced gene locations. The hot and cold categories derived from *K_A_/K_S_* are available as Dataset S1 and Dataset S2.

Selection on gene locations would provide an evolutionary explanation for the puzzle of why *K_A_* and *K_S_* are correlated beyond levels expected by neutral evolutionary theory (Mouchiroud et al. 1995; Ohta and Ina 1995). Assuming 4-fold sites are neutral, locational selection would have to be realized through the influence of the local mutation rate *K_S_* on the amino acid changing mutation rate *K_A_*. Thus, locational selection and point mutation-based amino acid selection would behave similarly with respect to positive or negative selection on protein sequence, increasing the correlation of *K_A_* and *K_S_*, even if mutations to any individual 4-fold site did not provide a fitness benefit.

One caveat is that other, not necessarily exclusive, explanations for the strong correlation of *K_A_* and *K_S_* have been proposed as well—most notably simultaneous substitutions at adjacent sites, so-called tandem substitutions (Smith and Hurst 1999b). Tandem substitutions were not sufficient to explain our hot and cold categories, however. We rederived sets of hot and cold categories after correcting for tandem effects (see Materials and Methods) and once again found similar results. For example, the six hottest categories (of 22 significant) were “dynein ATPase activity,” “receptor activity,” “homophilic cell adhesion,” “olfactory receptor activity,” “integral to membrane,” and “calcium ion binding activity.” The six coldest (of 36) were “nucleus,” “regulation of transcription, DNA dependent,” “RNA binding activity,” “transcription factor activity,” “development,” and “ribonucleoprotein complex.”

### Mechanisms

For the hot clustered categories, it may be that high mutation rates and high rates of gene duplication are tied to a hidden variable that imposes both phenomena simultaneously. One possibility is the recombination rate along the genome, which Pal and Hurst (2003) found to have selective effects in yeast. For example, two mechanisms for diversification, gene duplication and mutation, can both be accelerated by recombination (Graur and Li 2000; Lercher and Hurst 2002). High recombination rates are relevant for a number of the hot gene categories we have studied, as they have been suggested for the protocadherins (Wu et al. 2001), immune response (Papavasiliou and Schatz 2002), and olfactory families (Sharon et al. 1999). Because both gene duplication and point mutation are useful for diversifying a family, it is difficult to separate the significance of mutation rate and recombination rate. Pal and Hurst (2003) offered preliminary evidence that in yeast, selection acts on the recombination rate, but not point mutation rates. However, we have observed unusual rate biases for nonclustered gene categories as well, for which recombination would not be expected to play a role.

Cold gene categories are not clustered; therefore, the existence of cold categories (as well as nonclustered hot categories) cannot be attributed to duplication events. One alternate phenomenon that could cause cold category biases is gene relocation to cold regions. The concept of relocation brings up a number of questions. First, if cold genes have relocated, this leaves one wondering in what sort of environment cold genes originated. One speculative possibility is that these genes developed in regions of high recombination (the hot regions), which would have allowed for fast duplication and functional diversification, and later dispersed to cooler regions as their functions became fixed. Second, it is unclear whether gene relocations occur frequently enough to account for the observed rate biases. This issue is complicated by the fact that genes have arisen at different times. Many of the cold gene categories occur in diverse sets of tissues and have important regulatory effects, suggesting they should be relatively old. This old age may have allowed them enough time to redistribute through the genome.

We verified the correlations of substitution rates along the genome and showed that these correlations lead to an excess of hot and cold genes, confirming studies by Matassi et al. (1999) and Hardison et al. (2003). Our results appear to disagree with those of Kumar and Subramanian (2002), who reported that mutation rates are uniform in the genome. While our rate measurements were qualitatively similar to those of Kumar and Subramanian (2002), one beneficial addition we made was the use of a normalized rate that accounts for the length dependence of rate variance, allowing genes of differing lengths to be treated equally in Figure 1. Our correlation length analysis revealed two scales of rate correlation: a short decay length of 1 Mb and a long-range length extending along a syntenous block up to distances of 10 Mb. We have very speculatively proposed that DNA replication units and DNA recombination may be relevant to these length scales. More generally, it is hoped that these scale determinations will be helpful in placing constraints on possible processes that control mutation rate.

Some data issues suggest topics for further exploration. First, the resolution of our analysis is dictated by the structure of the GO taxonomy, which currently has 16,000 categories but is evolving. Our category inferences should become more specific as GO gene assignments improve. Second, multispecies data will be invaluable in revealing the mutations that have occurred in each lineage. One promising early result from human–chimpanzee comparisons, based on a set of 96 orthologs derived from HOVERGEN release 44 (Duret et al. 1994), is that olfactory receptors are a hot category. Unfortunately, this is the only statistically significant hot or cold category at present, owing to the lack of data. However, inferences should improve rapidly as more chimpanzee gene identifications become available.

## Materials and Methods

### 

#### Ortholog generation.

We downloaded a list of the available 37,347 human and 27,504 mouse peptides from the ENSEMBL sequence database (www.ensembl.org), then used BLAST (Altschul et al. 1990) to find orthologous peptide sequences between the genomes. The peptides studied were the set of all known or predicted peptides in the ENSEMBL 12.31.1 human and 12.3.1 mouse datasets. Sequences were designated as orthologous if the two peptides were each other's mutual best hit in the opposing databases, as determined by BLASTALL, and the E-value for the match was 10^−10^ (using the higher score as a worst-case bound) or better. We chose this method of ortholog determination to get a one-to-one relationship between proteins. We found 14,790 ortholog pairs, a coverage rate of approximately 50% in mouse and 40% in human. The observed E-values between orthologs have a median value of 0.0 (<1e − 180). The aligned peptide orthologs were then used in conjunction with ENSEMBL cDNA data to determine aligned orthologous cDNA. For the chimpanzee–human comparison, human genes from ENSEMBL were compared to chimpanzee genes from HOVERGEN. A mutual best-hit criterion was used to determine the set of 96 orthologs.

We manually inspected the mouse–human synteny of the olfactory gene cluster of Figure 4 to verify that orthologs were assigned correctly. This was to address the concern that orthologs are more difficult to assign in gene categories with many homologous members, since incorrect assignments could distort substitution rates. The synteny structure was found to be almost totally conserved for these genes, as it was for the cell adhesion genes in Figure 5.

#### Calculation of substitution rates.

We calculated the distribution of substitution rates between the mouse and human genomes using the 4-fold sites of orthologous genes; 4-fold sites are the third bases of codons for which the amino acid is specified by the first two bases. For each of the orthologous gene pairs, we calculated *p*, the fraction of 4-fold sites in which the mouse base differs from the human base. The average value of *p* over all 4-fold sites in all orthologs was <*p*> = 0.337. The average 4-fold substitution rate on a genewise basis was 0.338 with a standard deviation of 0.080. These rates were in agreement with substitution rates measured in other studies of 4-fold sites or in ancestral repeats (Mouse Genome Sequencing Consortium 2002; Hardison et al. 2003).

Because genes are of finite length, stochastic effects can cause substitution rates to vary from gene to gene, even if all 4-fold sites mutate at the same rate. We defined a normalized substitution rate to correct for these finite-size effects. A gene with *N* 4-fold sites was modeled as having *N *independent events in which substitution can occur with probability <*p*>. This formulation can fit both the Jukes–Cantor one-parameter or the Kimura two-parameter model for mutation matrices (Durbin et al. 1998). Although this model is not as sophisticated as other more modern treatments (e.g., see Tavere 1986; Tamura and Nei 1993; Li 1993; Goldman and Yang 1994), it gives an easily falsifiable prediction that the rate distribution, in the absence of regional correlation, can be approximated by a standard Normal distribution, due to the central limit theorem (Rice 1995).

Under this model, at each N the distribution of substitution rates can be described by a binomial distribution with a standard deviation of σ(*N*) = √<*p*>(1 − <*p*>)/*N* . Therefore, gene substitution rates were normalized by their respective σ(*N*) to get one universal rate distribution, which in the limit of many datapoints should approach the Normal distribution (2π)^−½^ exp (*x* ^2^/2). We defined the normalized substitution rate to be



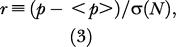
where *p* is the actual 4-fold substitution rate in the gene. The values of *r* for all ortholog pairs were used to calculate the distribution shown in Figure 1.

The actual rate distribution in genes was found to be skewed toward high or low mutation rates, as shown in Figure 1. The observed distribution had a standard deviation of 2.04, significantly higher than the expected σ = 1. Similar excesses of hot and cold genes were found even when corrections were made for base composition. To verify this, we calculated a normalized mutation rate using a four-parameter model in which each site of type A, C, G, or T in the human sequence has its own substitution probability. For each human base (A, C, G, and T), we measured the substitution rate at the corresponding 4-fold locations, yielding 4 rates <*p_A_*>, <*p_C_*>, <*p_G_*>, <*p_T_*>. Based on these rates, we then calculated the expected frequency and variance of substitutions for a gene given the gene's base composition at the 4-fold sites. This yielded a distribution nearly identical to that in the one-parameter model (see Figure 1).

We also tested whether neighboring genes have similar substitution rates. The orthologs were ordered by their location along the human genome, after which we calculated the Pearson correlation of a gene's substitution rate *r* with that of its following gene. We used only neighboring genes that were in syntenous blocks, as defined by all three conditions of monotonicity (the genes are ordered the same in both species), consistent strand orientation (a block is either in the same strand orientation in both species or completely reversed), and consistent chromosome (no chromosome changes in either species in a block), yielding a dataset of 11,087 neighbor pairs. Under this condition, the Pearson correlation was 0.26, corresponding to a highly significant *p*-value of 10^−189^.

#### *z*-score calculation for GO categories.

For each GO category, we calculated a normalized substitution rate (*z*-score) based on the substitution rates of all members of that category. Of the genes in our ortholog set, 9,966 had GO classifications available. The *z*-score was defined to be



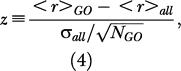
where <*r*>*_GO_* is the average substitution rate *r* for the genes in the GO category, <*r*>*_all_* is the average *r* for all of the genes with GO classifications, σ*_all_* is the genewise standard deviation, and *N_GO_* is the number of genes in the category. The *p*-value for *z* was determined from the probability that a Gaussian-distributed variable takes on a value ≥*z*. To reduce the problem of outliers, we limited our analysis to the GO categories containing at least five genes, of which there are 997, and accordingly set a *p*-value cutoff of 1/997 ∼ 10^−3^. We expressed the significance in terms of −log_10_ *p_z_*, which should have a value larger than 3 to be statistically significant.

*z*-scores corrected for tandem substitutions were calculated by first removing all possible tandem substitution sites from the dataset. That is, 4-fold sites were only accepted into the dataset if both the preceding and following bases matched in the two species. After culling the dataset, we calculated rates and category *z*-scores as before.

#### Clustering analysis.

To measure clustering, for each gene in a GO category we tested whether it had another category member downstream of it within the short-range correlation length of τ = 10^6^ basepairs. In each GO category, we calculated the number of genes satisfying this condition, defining this to be the number of “clustered genes.” This “downstream” criterion (rather than a symmetric one) was used to avoid the problem of double counting of genes when several are close together. To test the statistical significance of the number of clustered genes in a category, we used bootstrapping. For each GO category, we performed 5,000 random trials of selecting *N_GO_* random genes from the entire set of orthologs, where *N_GO_* is the number of genes in the GO category. In each trial, we counted the number of clustered genes in this randomly selected group. The average number of clustered genes was used to approximate the random number of clustered genes by a Poisson distribution. These Poisson statistics were then used to calculate the significance of the number of clustered genes for the GO category. A Poisson distribution is appropriate as long as clustering of neighbors is a rare event, i.e., as long as *N_GO_*<<*N_allgenes_*, which was generally the case.

The random distributions were visually inspected and found to agree with the shape of the Poisson curve. To generate the data for Tables 1 and 2, we also limited ourselves to the 997 categories with at least five genes, implying that −log_10_ *p_cluster_* > 3 is the cutoff for significance.

#### Calculation of repeat sequence mutation rates.

Aligned repeat sequences between mouse and human were obtained from the dataset of Hardison et al. (2003). For each repeat, positions in which a base was defined for both the mouse and human sequence were used to calculate a normalized substitution rate, in analogy with the method used for the 4-fold sites. The genome-wide average value of *p* in these repeat sequences was 0.33, which was very close to the value for 4-fold sites, 0.34. The start position of each repeat sequence was used to define its location in the genome. In order to determine the locations of repeat sequences (based on the June 2002 UCSC genome map) along the physical map used for the gene sequences (based on the ENSEMBL May 2003 map), gene locations according to the two maps were compared. Repeat sequence locations were then corrected using the location differences of nearby genes. For clarity, the ancestral repeat values shown in Figure 4 and Figure 5 were smoothed using a moving-window average of 20 repeat sequences.

#### Correlation length calculation.

We considered all pairs of genes on continuous orthologous blocks, starting from the first neighbor up to the 35th gene downstream. This allowed us to get hundreds of measurements of *r*(0)*r(t)* for *t* values even as large as several megabases. We binned these data into 100 uniformly spaced groups covering *t* ∈ [0, 15000000] and then averaged over each of these bins to determine the correlation function <*r*(0)*r(t)*>. The data were plentiful enough for the averaged values shown in Figure 6 to be statistically significant. It was difficult to extend to larger values of *t* since the amount of data decreases with *t*, a fact manifested in the increasing fluctuations at larger *t* in Figure 6. For example, the value of the average correlation <*r*(0)*r(t)*> at *t* = 15 Mb in the human data of Figure 6 was based on only 79 measurements, whereas at *t *= 75,000 it was based on 22,860 measurements. For genes with alternative splicings, only one of the genes was used, in order to avoid spurious effects caused by reuse of DNA. Orthologous block boundaries were defined by genes at which the chromosome changes in either species. Monotonicity and consistent strand orientation were ignored in order to obtain blocks with large values of *t*. Most of the *r*(0)*r(t)* data comes from blocks at least several megabases long. Approximately 5% is in blocks of size less than 10^6^ basepairs, 55% is in blocks of size between 10^6^ and 10^7^ basepairs, and the remaining 40% is in larger blocks.

The long-range correlation shown in Figure 6 was statistically significant. Theoretically, fluctuations in <*r*(*i*)*r*(*j*)> should be of the order ∼*O*(1/√*N* , where *N* is the number of data samples in a bin. At a distance of 10 Mb, there were approximately 400 samples, corresponding to an uncertainty of approximately 0.05. This uncertainty was an order of magnitude smaller than the observed value of *A*_∞_ = 0.4.

## Supporting Information

Dataset S1 Hot Gene Categories Based on *K_A_/K_S_*
Gene categories with significant positive selection on amino acid changing point mutations.
(23 KB XLS).

Dataset S2 Cold Gene Categories Based on *K_A_/K_S_*
Gene categories with significant negative selection on amino acid changing point mutations.
(21 KB XLS).

### Accession Numbers

The Gene Ontology (http://www.geneontology.org/) ID numbers for the categories discussed in this paper are as follows: brain development (GO:0007420), calcium-dependent cell adhesion molecule activity (GO:0008014), calcium-dependent protein serine/threonine phosphatase activity (GO:0004723), calcium ion binding activity (GO:0005509), carbohydrate metabolism (GO:0005975), cell adhesion (GO:0007155), cell growth and/or maintenance (GO:0008151), chymotrypsin activity (GO:0004263), CTD phosphatase activity (GO:0008420), cytoplasm (GO:0005737), development (GO:0007275), DNA binding activity (GO:0003677), dynein ATPase activity (GO:0008567), dynein complex (GO:0030286), enzyme activity (GO:0003824), G-protein coupled receptor protein signaling pathway (GO:0007186), GTP binding activity (GO:0005525), heterogeneous nuclear ribonucleoprotein (GO:0008436), homophilic cell adhesion (GO:0007156), immune response (GO:0006955), integral to membrane (GO:0016021), internalization receptor activity (GO:0015029), intracellular protein transport (GO:0006886), magnesium-dependent protein serine/threonine phosphatase activity (GO:0004724), membrane (GO:0016020), metabolism (GO:0008152), microtubule-based movement (GO:0007018), microtubule motor activity (GO:0003777), mRNA binding activity (GO:0003729), myosin phosphatase activity (GO:0017018), neurogenesis (GO:0007399), nucleus (GO:0005634), olfactory receptor activity (GO:0004984), oncogenesis (GO:0007048), protein amino acid dephosphorylation (GO:0006470), protein phosphatase type 2A activity (GO:0000158), protein phosphatase type 2B activity (GO:0030357), protein phosphatase type 2C activity (GO:0015071), protein serine/threonine kinase activity (GO:0004674), protein transporter activity (GO:0008565), proteolysis and peptidolysis (GO:0006508), receptor activity (GO:0004872), regulation of transcription, DNA-dependent (GO:0006355), regulation of transcription from Pol II promoter (GO:0006357), regulation of translational initiation (GO:0006446), ribonucleoprotein complex (GO:0030529), RNA binding activity (GO:0003723), RNA polymerase II transcription factor activity (GO:0003702), RNA splicing (GO:0008380), transcription coactivator activity (GO:0003713), transcription factor activity (GO:0003700), transcriptional activator activity (GO:0016563), translation regulator activity (GO:0045182), trypsin activity (GO:0004295), ubiquitin conjugating enzyme activity (GO:0004840), ubiquitin cycle (GO:0006512), and ubiquitin-dependent protein catabolism (GO:0006511).
